# Simple and Epididymal-Sparing Orchiectomy for Surgical Castration in Stage IV Prostate Cancer

**DOI:** 10.31486/toj.24.0013

**Published:** 2024

**Authors:** Harrison Travis, Michael Dubic, Joseph Bardot, Blane Edwards, Jessie R. Gills, Scott E. Delacroix, Stephen LaCour, Matthew Mutter, Donald Bell, Mary E. Westerman

**Affiliations:** ^1^ School of Medicine, Louisiana State University Health Sciences Center, New Orleans, LA; ^2^ Department of Urology, Louisiana State University Health Sciences Center, New Orleans, LA; ^3^ Department of Surgery, East Jefferson General Hospital, Metairie, LA

**Keywords:** *Castration*, *orchiectomy*, *prostate neoplasms*

## Abstract

**Background:** Androgen deprivation therapy, the mainstay of treatment for patients with advanced prostate cancer, can be either medical or surgical. Surgery has cost benefits compared to medical treatment. In this study, we evaluated the use of simple and epididymal-sparing orchiectomy in 2 different practice settings for the treatment of metastatic prostate cancer.

**Methods:** We reviewed patients who underwent surgical castration for metastatic prostate cancer between 2011 and 2022. The primary outcome was achieving castration-level total testosterone of <50 ng/dL. We also compared the characteristics of patients who were seen at a public academic teaching hospital vs those who were seen at a private community hospital (oncology group practice), and we evaluated the impact of orchiectomy approach (simple vs epididymal-sparing orchiectomy) on patient outcomes.

**Results:** We analyzed 101 patients who underwent orchiectomy: 40 (39.6%) at a public academic teaching hospital and 61 (60.4%) at a private community hospital (oncology group practice). Of these patients, 81 (80.2%) underwent simple orchiectomy and 20 (19.8%) underwent epididymal-sparing orchiectomy. Forty-nine patients (48.5%) had previously received medical androgen deprivation therapy, 9 (18.4%) of whom had medication adherence issues. Patient age, race, and marital status differed significantly between hospital facilities. The overall surgical complication rate was 3.0%. Postoperative total testosterone levels were available for 81 patients, drawn a median of 57 days after surgery [IQR 30, 123]. All patients had castrate-level total testosterone (median 10 ng/dL [IQR 9, 19]) postoperatively, with no differences seen between surgery location (*P* = 0.84) or surgical technique (*P* = 0.90).

**Conclusion:** Simple or epididymal-sparing orchiectomy is safe and effective for surgical castration and is an alternative to medical androgen deprivation therapy for patients diagnosed with metastatic prostate cancer regardless of the practice demographics.

## INTRODUCTION

In 2023, 288,300 males were expected to be diagnosed with prostate cancer, and 34,700 were predicted to die as a result of the disease.^1^ Lifelong androgen deprivation therapy is a treatment mainstay for metastatic prostate cancer and is aimed at preventing disease progression.^2^ Androgen deprivation therapy can be either medical via agents that inhibit the gonadotropin-releasing hormone in the hypothalamic-pituitary-gonadal axis or surgical via removal of the testicles. Individuals receiving systemic medical therapy for metastatic prostate cancer are typically treated with periodic injectable agents. Surgical androgen deprivation therapy is underutilized. Garje et al reported that in 2014, the majority of patients were treated with medical therapy and only 3.1% underwent orchiectomy according to the National Cancer Database.^3^ Orchiectomy has a cost advantage compared to using prescription medications for long-term (permanent) androgen deprivation therapy.^4,5^ A 2016 Canadian study of patients treated for prostate cancer from 1995 to 2005 found that the exclusive use of orchiectomy for androgen deprivation therapy (either as primary management or for metastatic disease) in 3,488 patients would result in first-year savings of $11,415,760, and savings would accrue to more than $75 million after 10 years compared to medical castration (prices in 2009 Canadian dollars).^5^ One concern with orchiectomy is the potential for psychological effects from changes in the physical appearance of the scrotum. However, research has shown that patients who undergo surgical management have fewer physical side effects and less disease-specific anxiety than medically managed patients.^6^

Multiple approaches to bilateral orchiectomy for permanent androgen deprivation therapy have been used: simple orchiectomy, subcapsular orchiectomy, and epididymal-sparing orchiectomy.^7,8^ All 3 techniques are efficacious in rapidly achieving a castrate-level total testosterone of <50 ng/dL, with some studies showing a time to castration level of as little as 3 hours to 12 hours postoperatively.^9,10^ Simple orchiectomy removes the entire testicle and spermatic cord, while the subcapsular and epididymal-sparing orchiectomy techniques preserve more of the scrotal contents, including the epididymis. A randomized trial by Bapat et al found that epididymal-sparing orchiectomy was associated with better aesthetic appearance compared to simple orchiectomy.^11^ Studies have reported that orchiectomy is as effective as medical castration in terms of postoperative patient satisfaction and survival, achieving castrate-level total testosterone levels, and decreasing prostate-specific antigen (PSA) levels.^7,12,13^

While national use of orchiectomy is low,^3^ our institution offers orchiectomy as an option for patients with stage IV prostate cancer, and we incorporated an epididymal-sparing orchiectomy technique in 2019.

In the outpatient setting, the current practice in our group is to initially offer both orchiectomy (a 30-minute procedure performed in the operating room under general anesthesia) and medical androgen deprivation therapy (injections every 6 months at a minimum) to patients with newly diagnosed advanced disease. Baseline PSA and total testosterone levels are obtained on all patients. Patients who choose orchiectomy and do not have a tissue diagnosis but a very high clinical suspicion of prostate cancer (PSA >100 ng/mL; presumed metastatic disease on imaging) undergo concurrent diagnostic prostate biopsy with frozen section confirmation. Postoperative laboratory workup is obtained 30 to 90 days following surgery, and most patients are started on an oral androgen receptor inhibitor in concordance with National Comprehensive Cancer Network (NCCN) guidelines.^14^ Nonsurgical patients are treated with dual agent androgen deprivation therapy in accordance with NCCN guidelines.^14^

Patients admitted to the hospital with de novo metastatic disease or advanced symptomatic disease are offered inpatient orchiectomy or initiation of medical therapy. These patients are referred to oncology for continued treatment following discharge.

To augment the real-world data on the use of orchiectomy, we describe the epididymal-sparing technique and provide information on short-term safety and efficacy outcomes in 2 distinct practice settings for patients who underwent simple or epididymal-sparing orchiectomy.

## METHODS

The institutional review board determined this study to be exempt (IRB #4320), and a waiver of informed consent was granted. Following institutional review board review, we retrospectively identified 163 patients who underwent orchiectomy (Current Procedural Terminology code 54520) from 2011 to 2022. Orchiectomy was performed by urologic surgeons either as an inpatient or outpatient procedure at 1 of 2 hospitals: a public academic teaching hospital and a private community hospital (oncology group practice). Patients who underwent orchiectomy for a diagnosis other than prostate cancer were excluded.

We extracted clinical and demographic features from the medical record, including the date and stage of the initial prostate cancer diagnosis and prior treatment including the use of medical androgen deprivation therapy. We reviewed notes for issues related to patient adherence, therapy interruption, or gaps in care. We reviewed operative reports to determine the type of orchiectomy performed (simple orchiectomy or epididymal-sparing orchiectomy), as well as any additional procedures. Intraoperative and 30-day postoperative complications were recorded.

Presurgical PSA and total testosterone levels were recorded from the most recent laboratory data prior to the date of surgery. Postsurgical PSA and total testosterone values and date drawn were also recorded. The first laboratory values following surgery were used, typically drawn 30 days to 90 days postoperatively. For levels reported as <[value], the closest whole number was used for analysis. For example, total testosterone <10 ng/dL was analyzed as a total testosterone of 9.0 ng/dL. Some patients did not have postoperative total testosterone levels available, but they were included in the analysis of surgical complications.

Distance to the hospital and median household income were determined with the CDXStreamer (Hughes Financial Services Inc) batch report using patient and hospital ZIP codes.

The primary study outcome was achieving castrate-level total testosterone of <50 ng/dL. We also compared the demographic and clinical characteristics of the patients seen at the 2 facilities, as well as the impact of surgery location and orchiectomy approach on patient outcomes.

### Surgical Techniques

For patients in the simple orchiectomy cohort, a single vertical midline incision is made in the median raphe through the tunica vaginalis. The testicle is delivered, and the cord is separated into 2 bundles. Kelly clamps are used to clamp the spermatic cord bundles. Sharp division is made, and 2-0 Vicryl ties are placed proximally and distally. This step is repeated for the remaining bundle, and the remaining proximal cord is replaced in the scrotum. These steps are repeated for the contralateral testicle. Hemostasis is confirmed, and the dartos muscle is reapproximated with interrupted 3-0 Vicryl sutures. Skin edges are closed with interrupted 4-0 chromic suture and 25% bupivacaine.

**Figure 1.**
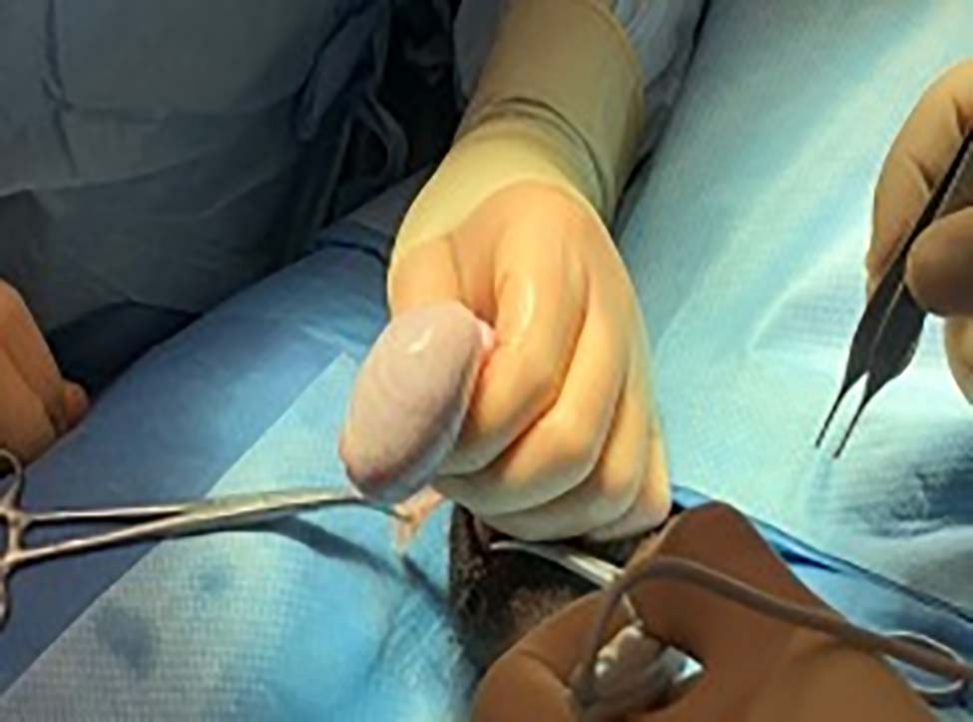
The testicle is delivered through a small scrotal incision.

**Figure 2.**
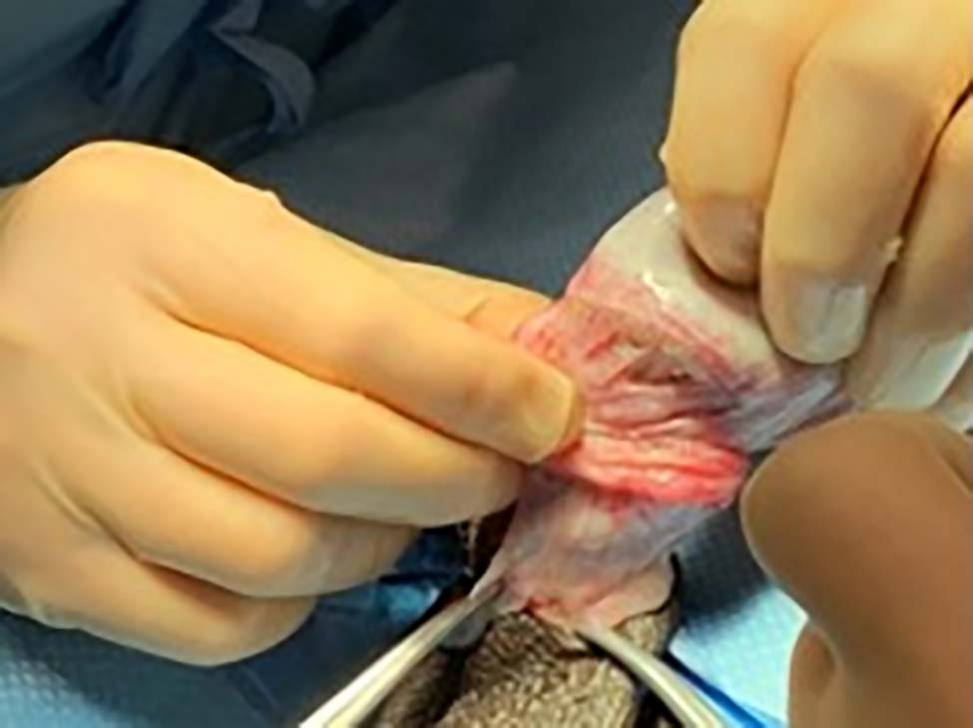
The parietal layer of the tunica vaginalis is peeled back.

**Figure 3.**
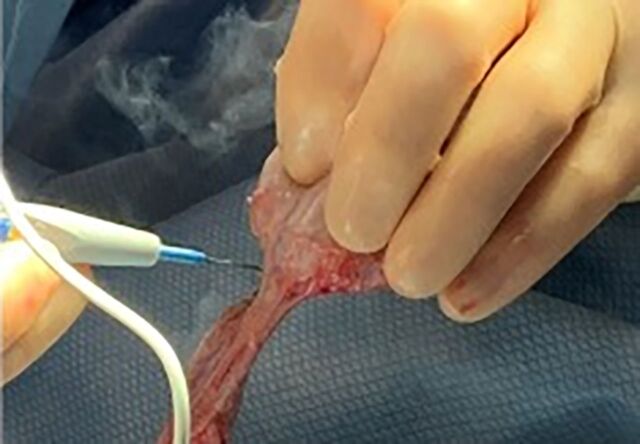
The head and tail of the epididymis with efferent ductules are dissected from the tunica albuginea using cautery.

**Figure 4.**
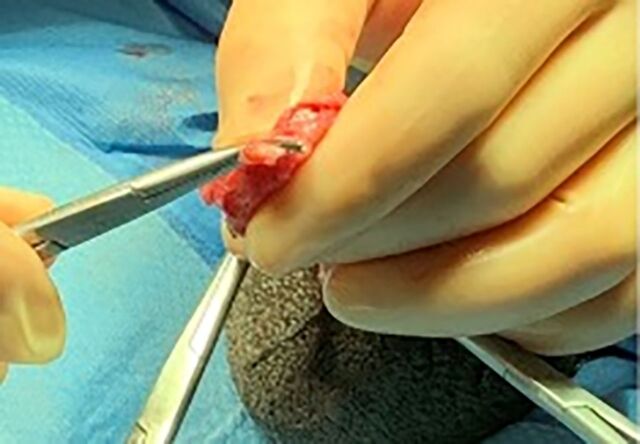
Perforating branches of the testicular artery are either suture tied or ligated with electrocautery.

**Figure 5.**
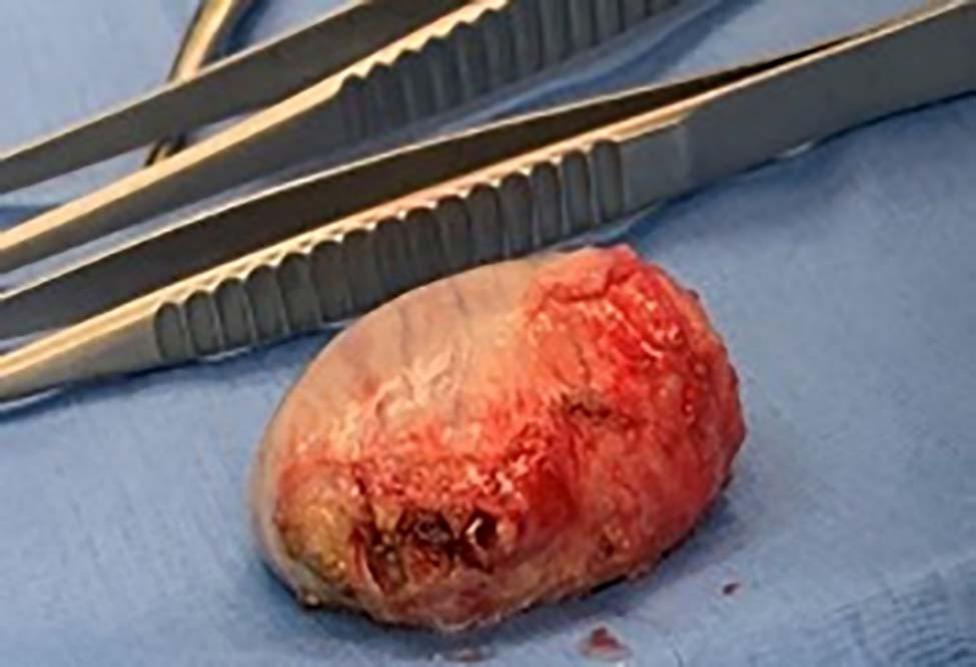
The testicular hilum is controlled with electrocautery.

**Figure 6.**
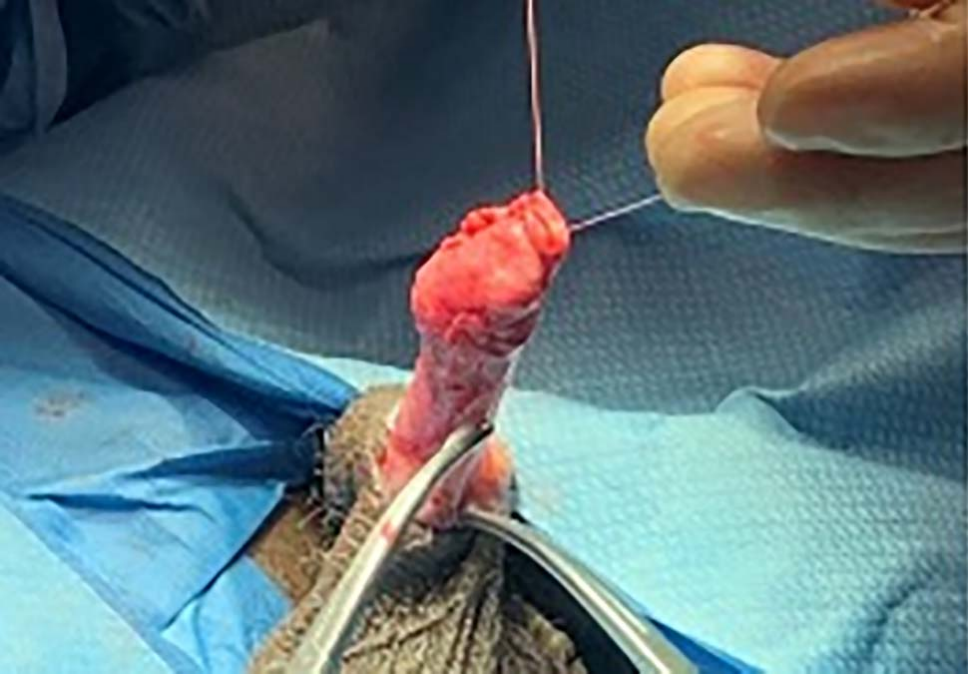
The tail of the epididymis is approximated to the head (shown) and then placed back inside the tunica vaginalis, which is sutured, and the incision is closed.

The epididymal-sparing orchiectomy technique used by 2 urologic oncologists (JRG and MEW) is illustrated in Figures 1 to 6. A 2-cm incision is made in the hemiscrotum, and the testicle is delivered (Figure 1). The tunica vaginalis is divided and preserved (Figure 2). The epididymis with efferent ductules is dissected from the tunica albuginea using cautery (Figure 3). The main testicular blood supply is either suture tied or ligated with electrocautery (Figure 4). The testicular hilum is controlled with electrocautery (Figure 5). Once freed from the epididymis, the testicle is removed from the surgical field. The body of the epididymis is inspected for hemostasis. Figure 6 illustrates a 2-0 Vicryl SH needle being used to bring the head, body, and tail of the epididymis together, forming a small nubbin. The epididymis is placed inside the tunica vaginalis which is then closed with a running 3-0 Vicryl suture. The dartos is closed, followed by skin closure. The same procedure is repeated on the opposite side.

### Statistical Analysis

Continuous features are summarized using medians and interquartile ranges [IQR], while categorical features are summarized with frequency counts and percentages. The significance of differences across groups was assessed with the chi-square test for categorical variables and the Wilcoxon rank-sum test for continuous variables. The statistical program JMP 16.1.0 (SAS Institute Inc) was used for this study. All tests were 2-sided, with *P* <0.05 considered statistically significant.

## RESULTS

Between 2011 and 2022, 163 males underwent orchiectomy at the 2 facilities, and 101 met the inclusion criteria for this study. Baseline features are shown in Table 1: 71.3% had stage IV disease at the time of diagnosis; 48.5% had previously received medical androgen deprivation therapy, with the predominant therapy being luteinizing hormone-releasing hormone (LHRH) agonist (51.0%, 25/49); and 18.4% (9/49) of the patients who had received medical androgen deprivation therapy had documented lapses in treatment. Six of the 9 patients had lapses in treatment because of difficulties paying for medical treatment: 3 could not afford the co-payments, 2 lost their insurance, and 1 had a breakthrough total testosterone level of 514 ng/dL while adherent with LHRH agonist but was unable to afford abiraterone. The other 3 of the 9 patients had documented gaps in care for their prostate cancer of 3 years to 5 years during which they received no treatment. Most patients (88.9%, 8/9) with adherence issues were seen in the oncology group practice (*P* = 0.0001).

**Table 1.** Demographic and Clinical Characteristics of Patients Overall and by Surgery Location, n = 101

Variable	All Patients, n = 101	Public Academic Teaching Hospital, n = 40	Private Community Hospital (Oncology Group Practice), n = 61	*P* Value
Age, years, median [IQR]	70 [63.5, 79.5]	66 [61, 72]	74 [64, 82]	0.0015
Inpatient	15 (14.9)	10 (25.0)	5 (8.2)	0.025
Outpatient	86 (85.2)	30 (75.0)	56 (91.8)	0.02
Orchiectomy type				<0.0001
Simple	81 (80.2)	39 (97.5)	42 (68.9)	
Epididymal-sparing	20 (19.8)	1 (2.5)	19 (31.1)	
Race				<0.0001
Black	50 (49.5)	32 (80.0)	18 (29.5)	
White/other	51 (50.5)	8 (20.0)	43 (70.5)	
Marital status				0.004
Married	49 (48.5)	12 (30.0)	37 (60.7)	
Other	52 (51.5)	28 (70.0)	24 (39.3)	
Distance from facility, miles, median [IQR]	23.8 [15, 62]	15.1 [4.3, 66.7]	25.9 [17, 57]	0.08
Household income per ZIP code, dollars, median [IQR]	56,167 [41,046, 65,229]	52,775 [40,019, 60,595]	56,361 [43,627, 65,501]	0.14
Stage IV at diagnosis	72 (71.3)	29 (72.5)	43 (70.5)	1.0
Prior definitive local therapy	27 (26.7)	10 (25.0)	17 (27.9)	0.8
Prior medical androgen deprivation therapy	49 (48.5)	23 (57.5)	26 (42.6)	0.14
Noncompliance with medical androgen deprivation therapy^a^	9/49 (18.4)	1 (11.1)	8 (88.9)	0.0001
Concomitant procedure	26 (25.7)	7 (17.5)	19 (31.1)	0.11
Prostate biopsy	14 (53.8)	3 (42.9)	11 (57.9)	
Relief of urinary tract obstruction	8 (30.8)	2 (28.6)	6 (31.6)	
Other	4 (15.4)	2 (28.6)	2 (10.5)	
Surgical complication	3 (3.0)	2 (5.0)	1 (1.6)	0.56
Clavien-Dindo II (wound infection)	2 (66.7)	2 (100)	0	
Clavien-Dindo IIIb (return to OR for bleeding)	1 (33.3)	0	1 (100)	

^a^Percentages for the individual groups are calculated across the row with a denominator of 9.

Note: Data are presented as n (%) unless otherwise indicated.

IQR, interquartile range; OR, operating room.

The majority of patients (80.4%) underwent simple orchiectomy, with 19.6% undergoing epididymal-sparing orchiectomy. Most patients (85.2%) underwent outpatient surgery, with inpatient procedures significantly more likely to occur at the public academic teaching hospital than at the private community hospital (oncology group practice) (25.0% vs 8.2%, respectively, *P* = 0.025).

Twenty-six patients (25.7%) had concomitant procedures such as prostate biopsies or relief of urinary tract obstruction. The overall surgical complication rate was 3.0%, and all 3 complications occurred in the simple orchiectomy group: 2 Clavien-Dindo Grade II complications (wound infections) and 1 Clavien-Dindo Grade IIIb complication (return to the operating room for spermatic cord bleeding).

### Demographics

In the public academic teaching hospital practice, patients were younger (median age of 66 years), and more identified as Black (80.0%) compared to the private community hospital (oncology group practice) group where the median age was 74 years and most patients identified as White/other (70.5%). Marital status was significantly different between the groups: 60.7% of patients in the private community hospital (oncology group practice) group were married vs 30.0% in the public academic teaching hospital group (*P* = 0.004). Distance to the facility and household income did not differ significantly between groups, nor did factors related to prior diagnosis or treatment aside from the documented adherence issues reported in the previous section.

### Castration Levels

Table 2 shows the median preoperative and postoperative total testosterone and PSA levels, stratified by surgery location and by orchiectomy type. No significant differences in preoperative total testosterone and PSA levels were seen between the simple and epididymal-sparing orchiectomy groups.

**Table 2.** Surgical Complications, Preoperative and Postoperative Total Testosterone and Prostate-Specific Antigen (PSA) Levels, and Median Days Until Postoperative Laboratory Work Overall and by Surgery Location and Orchiectomy Type

		Surgery Location			Orchiectomy Type		
Variable	All Patients, n = 101	Public Academic Teaching Hospital, n = 40	Private Community Hospital (Oncology Group Practice), n = 61	*P* Value	Simple, n = 81	Epididymal-Sparing, n = 20	*P* Value
Surgical complication	3 (3.0)	2 (5.0)	1 (1.6)	0.57	3 (3.7)	0	1.0
Preoperative total testosterone available	61 (60.4)	15 (37.5)	46 (75.4)	0.0006	46 (56.8)	15 (75.0)	
Preoperative total testosterone, ng/dL, median [IQR]	206 [20, 332.5]	32 [9, 269]	225 [27, 373]	0.02	207 [20, 331.5]	172 [12, 339]	0.99
Postoperative total testosterone available	81 (80.2)	32 (80.0)	49 (80.3)	0.96	67 (82.7)	14 (70.0)	0.20
Postoperative total testosterone, ng/dL, median [IQR]	10 [9, 19]	9 [9, 22.5]	12 [9, 19]	0.84	12 [9, 19]	9 [9, 11.5]	0.90
Patients on medical ADT who were castrate prior to orchiectomy, n = 49	17/49 (34.7)	8/23 (34.8)	9/26 (34.6)	0.99	15/43 (34.9)	2/6 (33.3)	0.84
Preoperative PSA, ng/mL, median [IQR], n = 101	58.4 [8, 378]	124.2 [10.5, 1115.5]	34.2 [7.2, 141.3]	0.04	73.4 [8, 378]	28.9 [9.4, 466]	0.64
Postoperative PSA ng/mL, median [IQR], n = 77	2.45 [0.3, 17.2]	6.4 [0.4, 107.15]	1.7 [0.3, 6.1]	0.05	2.45 [0.2, 17.2]	2.65 [0.98, 32.6]	0.38
Days to postoperative laboratory work, median [IQR]	57 [30, 123]	43.5 [23, 78]	70 [40, 177]	0.0003	65 [32, 125]	45 [26, 80]	0.36

Note: Data are presented as n (%) unless otherwise indicated.

ADT, androgen deprivation therapy; IQR, interquartile range; PSA, prostate-specific antigen.

Eighty-one (80.2%) patients had postoperative testosterone levels available (Table 2). At a median of 57 days, all patients with available laboratory work reached castrate-level total testosterone <50 ng/dL. Patients in the epididymal-sparing orchiectomy group had a lower median postoperative total testosterone level (9 ng/dL) than patients in the simple orchiectomy group (12 ng/dL); however, this difference did not reach statistical significance (*P* = 0.90).

The median postoperative PSA level for all patients was 2.45 ng/mL, decreased from 58.4 ng/mL preoperatively, and no significant differences were found between the simple orchiectomy and epididymal-sparing orchiectomy groups in either the preoperative median levels of PSA (73.4 ng/mL vs 28.9 ng/mL, respectively) or the postoperative median levels of PSA (2.45 ng/mL vs 2.65 ng/mL, respectively).

## DISCUSSION

Surgical castration is a viable yet underutilized option for long-term androgen deprivation therapy in males with metastatic prostate cancer.^3^ This study adds support to the existing literature that orchiectomy is effective, as all patients obtained castrate-levels of total testosterone by a median of 57 days postoperatively. We also show that orchiectomy is accepted by a wide demographic of patients as demonstrated by use of the procedure at 2 facilities treating different patient populations. Importantly, this technique appears to be safe, ensures adherence, and can be implemented into general urologic practices.

When counseling patients about orchiectomy, care should be taken to avoid using terminology that may portray orchiectomy as a less efficacious or more emasculating option than medical androgen deprivation therapy. When explaining the epididymal-sparing orchiectomy, we inform patients that only the part of the testicle that produces testosterone and sperm will be removed, and that all other structures will be left in place and closed to resemble a regular testicle. As mentioned previously, this option is presented at the initial clinic visit and can be revisited later if the patient chooses to start with medical androgen deprivation therapy initially.

In a retrospective study of 10,675 patients with metastatic prostate cancer from the California Cancer Registry, Borno et al found that Hispanic males and males who lived in neighborhoods with a lower socioeconomic status or had Medicaid/public insurance were more likely to undergo orchiectomy.^15^ In our study, while significant differences in patient race and marital status were seen between hospital facilities, they are likely attributable to differences between the general patient populations that these sites serve and not related to patient willingness to undergo surgery. In addition, patients at the public academic teaching hospital were more likely to undergo simple orchiectomy, which may be related to the supervising surgeon. Larger studies investigating the frequency and clinical presentation of surgical castration, as well as patient perceptions of surgery, may be warranted. The optimal method of discussing surgical castration may need to be tailored to the individual patient by incorporating cultural and personal values and beliefs.

In our study, 12.2% (6/49) of patients who underwent orchiectomy after a period of medical androgen deprivation therapy had medication adherence issues because of documented insurance issues or costs. Studies have demonstrated inconsistent administration and documentation of treatment plans in clinical practice for patients receiving medical androgen deprivation therapy.^16,17^ Crawford et al retrospectively found that 84% of androgen deprivation therapy injections were administered late, beyond the 28-day month definition used in clinical trials.^16^ Patients who received injections 2 weeks late had total testosterone levels 4 times greater than patients whose injections were administered on time (98 ng/dL vs 21 ng/dL, respectively).^16^ In a single-center retrospective study, Bochner et al found that 55% of patients undergoing androgen deprivation therapy did not have a documented plan of care, and 7.1% of patients had a documented missed dose.^17^ While we do not know the rates of nonadherence among our patients on medical androgen deprivation therapy who did not undergo orchiectomy, studies evaluating the prevalence of nonadherence and identifying the barriers to adherence are needed.

The cost benefit of orchiectomy compared to medical androgen deprivation therapy has been demonstrated.^4,5^ In 2016, Weinberg et al examined costs for epididymal-sparing orchiectomy and found that the cost of the operation performed in the operating room under general anesthesia was equivalent to the cost of 4 months of leuprolide and to 7.8 months of degarelix.^4^ Costs were further decreased when the procedure was performed under conscious sedation. Our surgeries were performed under general anesthesia.

The overall surgical complication rate in our study was 3.0%, and all complications occurred in the simple orchiectomy group. Rud et al reported a complication rate of 14.4% in a study of 83 patients who underwent subcapsular bilateral orchiectomy, partly attributed to resident surgeon inexperience.^12^ Other studies reported few or no complications in patients undergoing epididymal-sparing orchiectomy under local anesthesia with or without conscious sedation.^4,7,9^ The advantage of epididymal-sparing orchiectomy compared to simple orchiectomy is better preservation of the scrotal contents, resulting in less negative psychological effects and better overall patient satisfaction.^9,18,19^ Issa et al described a subset of patients who underwent epididymal-sparing orchiectomy after a period of medical androgen deprivation therapy; the patients reported high satisfaction with the procedure and said they preferred surgical over medical intervention.^7^ In the studies by Potosky et al and Atta et al, patients who received medical androgen deprivation therapy and then underwent orchiectomy had better self-reported outcomes because of less concern about insufficient medical management, fewer androgen deprivation therapy–related side effects, and a better feeling of overall health.^6,20^

The preoperative and postoperative total testosterone and PSA levels in the patients in our study match what has been described.^7,13,20^ While we used the accepted definition of castration (total testosterone <50 ng/dL) for this study, evidence supports that lower total testosterone levels (<20 ng/dL) provide improved survival and time to disease progression.^21,22^ Atta et al showed that orchiectomy resulted in lower total testosterone levels than medical therapy.^20^ Conversely, Østergren et al found that medical castration with LHRH agonists led to a greater proportion of patients achieving total testosterone <20 ng/dL compared to those who underwent subcapsular orchiectomy.^23^ However, all patients in the Østergren et al study, regardless of treatment, had a total testosterone level <30 ng/dL, with 79% of the orchiectomy patients reaching total testosterone levels <20 ng/dL within 12 weeks and 87% at 48 weeks. Unlike our study, the Østergren et al patient cohort was androgen deprivation therapy–naïve with a recent diagnosis of advanced prostate cancer.^23^ In our study, 100% of patients who underwent orchiectomy had total testosterone <50 ng/dL at a median 57 days after orchiectomy.

### Limitations

Our data are limited by the retrospective nature of the study and the relatively small sample size, particularly for the epididymal-sparing orchiectomy technique. In addition, some patients did not have preoperative and/or postoperative laboratory values available, generally because of inconsistency in practice patterns between the 2 sites. We did not gather data on long-term survival, specifically any cardiac-related morbidity following surgery. We also do not have quality of life or patient satisfaction data. However, the results of this study affirm the effectiveness of the orchiectomy procedure.

## CONCLUSION

Surgical castration is a feasible and effective treatment for metastatic prostate cancer. In this real-world study, acceptance of surgical castration for stage IV prostate cancer was not restricted to patients with particular demographics. Consistently and appropriately offering surgical androgen deprivation therapy as an alternative to medical androgen deprivation therapy to patients with advanced prostate cancer should be a part of urologic practices that treat these patients.

## Notes

*This article meets the Accreditation Council for Graduate Medical Education and the American Board of Medical Specialties Maintenance of Certification competencies for Patient Care, Medical Knowledge, and Practice-Based Learning and Improvement.*
